# A Three-Dimensional Analysis of Morphological Variations in Maxillary Second Molar in a North Indian Population Using Cone-Beam Computed Tomography

**DOI:** 10.7759/cureus.27086

**Published:** 2022-07-20

**Authors:** Nahid Afzal, Aishwarya Sinha, Navneet Kaur, Mohit Yadav, Vikram Pal Aggarwal, Aditi Sharma

**Affiliations:** 1 Department of Conservative Dentistry and Endodontics, Subharti Dental College, Meerut, IND; 2 Department of Orthodontics & Dentofacial Orthopedics, Subharti Dental College, Meerut, IND; 3 Public Health Dentistry, Surendera Dental College & Research Institute, Sriganganagar, IND; 4 Department of Public Health Dentistry, Swami Devi Dyal Hospital and Dental College, Panchkula, IND

**Keywords:** root canal therapy, endodontics, canal morphology, maxillary second molar, cone beam computed tomography

## Abstract

Background: Cone beam computed tomography (CBCT) has evolved in the field of endodontics and has helped to diagnose and treat the case very easily and accurately. The researchers set out to pinpoint the exact placement of the roots and canals in the maxillary second molars of North Indians by analyzing CBCT pictures.

Methods: In this study, in vivo CBCT was used to examine the maxillary second molars (n = 70) in detail. Both the number and configuration of root canals may be determined using Vertucci's categorization.

Results: Most people had three roots in their second molars (85.7%). Most maxillary second molars that had three roots looked like they had three separate roots (81.7%). In the roots of 85.7% of maxillary second molars, one canal was found in the mesiobuccal roots, and 14.2% had an MB2 canal. All of the canals in the palatal, distobuccal root, and MB1 root were Type I. The Type II canal configuration was found in 11.7% of MB2 canals. Type IV canals were found in 5% of the MB2 canals. The number of maxillary second molars with MB2 was found to be the same for both men and women (P =0.11). The number of MB2 cases did not depend on where the teeth were or how old the person was (P=0.08 and 0.06, respectively). The fact that both second molars appeared at the same time was important (P<0.001).

Conclusions: We report the occurrence of unusual morphologic abnormalities that affect only one root and have only been described in case reports. CBCT scans can help doctors better understand root canal anatomy and potentially enhancing endodontic management outcomes.

## Introduction

Root canal therapy's primary goal is to clean, seal and remove any debris from the root canal. These aims can only be attained with a basic understanding of root canal therapy, which dentists require in order to help their patients [1]. The frequency of various root canal designs and the existence of anatomical differences should be known by clinicians [2]. Evaluation of root canal morphology in vivo has traditionally relied on intraoral periapical radiography [3]. Despite the fact that radiographs depict things in three dimensions (3D), the missing dimension causes the structures to seem distorted and layered. The use of cone-beam computed tomography (CBCT) images for diagnostic and treatment planning prior to beginning endodontic therapy has proven to be beneficial [4,5].

It has been built specifically for use in dentistry to utilize cone-shaped X-ray beams rather than fan-shaped beams. The non-invasive nature of CBCT and the possibility of 3D root reconstruction are the key advantages of this technology. CBCT scans are better for clinical usage because they utilize less radiation, take less time (10-15 seconds), cost less money, and have better precision and resolution than standard spiral computed tomography scans [6]. CBCT has been indicated as a useful imaging tool in cases where root canal systems need to be located and interpreted in complex situations [7]. Root anatomy and canal morphology of maxillary molars have been extensively studied because of their complexity. Published evidence indicates that most maxillary molars have three roots and four canals. Studies consistently reveal that around half of all mesiobuccal (MB) roots have a second canal, mesiobuccal 2 (MB2) [1,8-11].

Root canal configurations vary in various racial populations. This study has been designed for analysis of maxillary second molars in North Indian people using CBCT. Hence the purpose of this research was to use CBCT scanning to examine root and root canal abnormalities in maxillary second molars.

## Materials and methods

This cross-sectional double-blind in vitro investigation was conducted between July 2018 and June 2019. The project began when the Institutional Ethical Committee gave its assent with IRB number SDC/IEC/2022/026. In Meerut, 70 CBCT images of maxillary second molars were evaluated.

**Figure 1:**
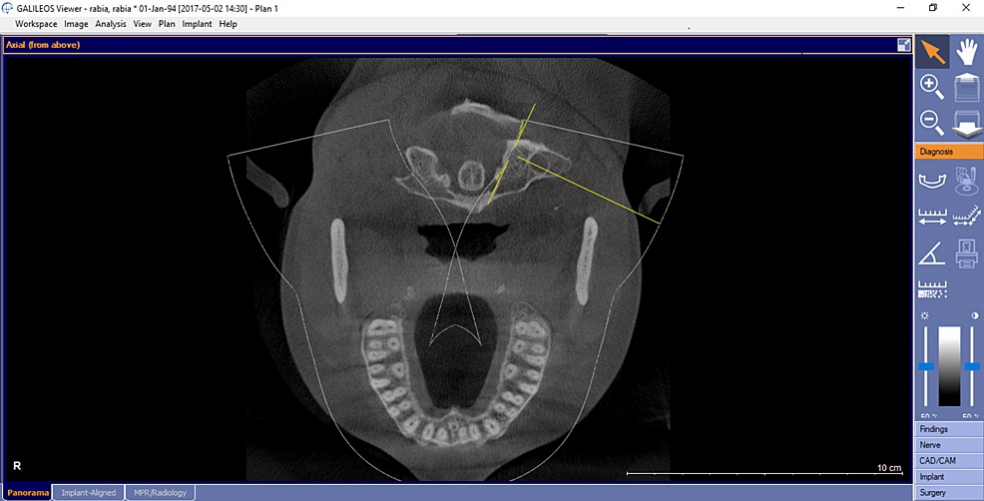
Cases of maxillary second molar with three roots

**Figure 2:**
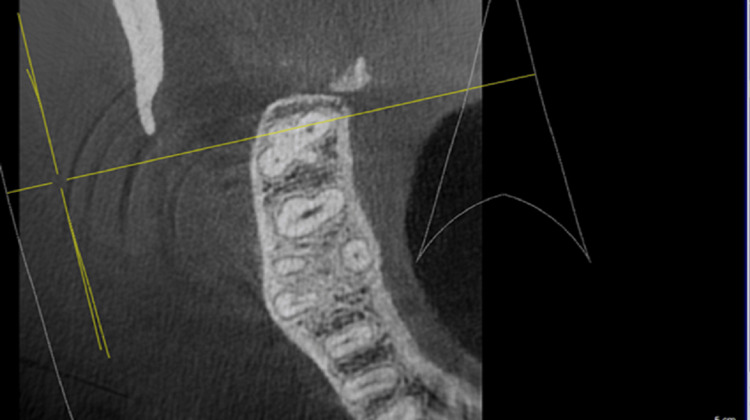
Cases of maxillary second molar with single root

**Figure 3:**
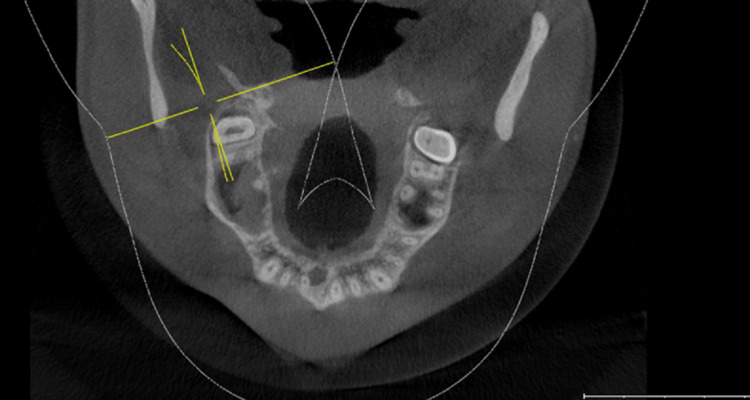
Cases of maxillary second molar with two roots

Subjects who had CBCT scanning for various diagnostic objectives were used in our study. There was no research-related exposure for any of the patients in this study. The department of oral medicine and radiology's pre-existing database was used to acquire all of the data. On a 32-inch Dell LCD panel with a resolution of 1280x1080, the CBCT data was analyzed using Galaxis and Sidexis software (Dentsply-Sirona Implants, York, PA, USA) . Axial, sagittal, and coronal sections were all examined (Figures 1-3).

When necessary, the software's contrast, brightness, and magnifying functions were employed and changed. If confirmation couldn't be found, an oral radiologist was asked for a second opinion. The number of roots, canals, and canal features in each tooth in each image of a maxillary molar was meticulously counted. Vertucci's and Gulabiwala's classifications were developed based on root canal configuration.

Statistical analysis

Our study was performed using SPSS software, version 20.0 (IBM Corp., Armonk, NY, USA). Information collected from the categorical variables was shown as frequencies and percentages. Statistical relationships between categorical variables were examined using chi-square analysis. The findings were statistically significant at the 0.05 level of significance.

## Results

Most people had three roots in their second molars (85.7%). Most maxillary second molars that had three looked like they had three separate roots (81.7%) (Table 1).

**Table 1:** Numbers and morphology of roots in maxillary second molars

Number and morphology of roots	N	%
One conical root	2	2.8
Two roots	8	11.5
Two separate roots (2S)	7	87.5
Two fused roots (2F)	1	12.5
Three roots	60	85.7
Three separate roots (3S)	49	81.7
Two fused one separate root (2F1S)	8	13.3
Three fused roots (3F)	3	5

The present study did not observe any four-rooted maxillary second molars. Only one canal was found in the palatal root of all three roots of the maxillary second molar. All three maxillary second molars with three roots were found to have one canal in the distobuccal root. In the roots of 85.7% of maxillary second molars, one canal was found in the mesiobuccal roots, and 14.2% had an MB2 canal (Table 2).

**Table 2:** Number of canals in maxillary second molars with three roots

Root canals	Number of canals	
	One	Two
Palatal root, % (N)	100% (0)	0
Distobuccal root, % (N)	100%(0)	0
Mesiobuccal root, % (N)	85.7% (50)	14.2% (10)

The maxillary second molar canals were analyzed based on Vertucci's categorization system. It was determined that the roots and canals of teeth with all their roots connected were too complicated to categorize. We were provided with two single-rooted maxillary second molars. Single-rooted maxillary second molars may have a variety of canal configurations, with Type 1 (1) and Type IV (2) being the most common. A total of eight maxillary second molars were found to have split roots. Eight maxillary second molars had a Type I (2) canal structure, both at the buccal and palatal root ends (Table 3).

**Table 3:** Configuration of root canal system in maxillary second molars B: Buccal, P: Palatal, DB: Distobuccal, MB: Mesiobuccal, Data is presented as N (%)

No of roots	Roots	Type I (1)	Type II (2-1)	Type III (1-2-1)	Type IV (2)	Type V (1-2)	Type VI (2-1-2)	Type VII (1-2-1-2)	Type VIII (3)
Single root (n=2)		1 (50)	0	0	0	1(50)	0	0	0
Two roots (n=8)	Buccal, Palatal	8 (100)	0	0	0	0	0	0	0
Three roots (n= 70)	Palatal, Distobuccal, Mesiobuccal 1, Mesiobuccal 2	60 (100)	7 (11.7)	0	3 (5)	0	0	0	0

Sixty maxillary second molars were presented with three root morphology. All the palatal, distobuccal root and MB1 root presented with Type I canal configuration. In 11.7% of MB2 canals, Type II canals were found. In 5% of MB2 canals, Type IV canals were found.

Table 4 shows the frequency of additional MB root canals (MB2) by gender, tooth position (right or left), and age. The frequency of MB2 in maxillary second molars did not differ statistically significantly between sexes (P=0.11). Because of this, neither tooth location nor age was associated with an increased risk of MB2 infection (P=0.08 and 0.06 respectively).

**Table 4:** Number and frequency of subsequent root canals in mesiobuccal (MB) roots of maxillary second molars evaluated by sex, tooth position and age P value less than 0.05 is considered statistically significant

Parameters	N (%)	P value
Sex		
Male	6/10 (60)	0.11
female	5/10 (50)	
Tooth position		
Left	5/10 (50)	0.08
Right	5/10 (50)	
Age		
10 – 19	1/10 (10)	0.06
20 – 29	4/10 (40)	
30 – 39	3/10 (30)	
40 – 49	1/10 (10)	
>50	1/10 (10)	

There were a total of 70 pairs of maxillary second molars in which MB2 was found to be present (from 35 patients). MB2 bilateral molars are more common in the contralateral molars, as evidenced in Table 5 by their frequency of co-occurrence. P=0.001 was the significance level for the bilateral second molar pairs that had this contemporaneous appearance.

**Table 5:** Unilateral and bilateral occurrence of additional root canals in mesiobuccal (MB) roots among 35 patients with bilateral maxillary second molars P value <0.05 is considered significant

Tooth	MB root with additional root canal			MB root with one canal	Odds ratio 95% CI	P value
	Unilateral		Bilateral	Bilateral		
	Left N (%)	Right N (%)	N (%)	N (%)		
Maxillary second molar	2 (5.7)	2 (5.7)	3 (8.6)	28 (80)	18.22 (10.12 –32.44)	<0.001*

## Discussion

Proper endodontic treatment requires an in-depth familiarity with the anatomical morphology and variability of the maxillary molars. Root canal systems often include many branches due to the canal's tendency to split and then re-join. There are four major types of root canals according to Weine et al. [2]. More complex canal systems were found by others, but Vertucci was the first to uncover an array of eight distinct designs for canal spaces [1].

Some of the most commonly used techniques and procedures include staining of the root canal, cross-sectioning and contrast medium enhanced radiography [12,13], radiographic evaluation [14,15], and computerised tomography scanning.

Although canal staining, cleaning, and cross sectioning techniques are disruptive, they always result in changes. Furthermore, intraoral periapical radiography has the drawback of only producing two-dimensional pictures. All of these methods have significant limitations that make it impossible to reliably assess the intricacy of a root canal's architecture. CBCT's ability to offer vital information in three dimensions has made it more popular in the area of endodontics as a non-invasive approach for analyzing the external and interior morphology of the root and root canal systems [16-18].

CBCT may take images with slice thicknesses ranging from 90 millimeters to 300 millimeters. Our analysis used slices that were 300 mm in thickness. Moreover, because the voxels (3D pixels containing information) in CBCT images are isotropic, the calculations are accurate from a purely mathematical standpoint. Some needs are met by CBCT exams, such as diagnosis, treatment planning, evaluation of intraoral pathologies, evaluation of root canal morphology and shape, evaluation of root resorption components, evaluation of the concept of obturation and assistance with the removal of root canal fillings, preoperative preparation, and evaluation of internal and external root resorption. Some research suggests that the second mesiobuccal channel of human maxillary molars may be identified using CBCT [19,20].

Intraoperative CBCT imaging is an excellent alternative that can be utilised in the event that an unexpectedly complicated anatomy is observed during access or if canals are not detected. CBCT can address one of the most critical issues for an endodontist - how many canals there are in each root. We discovered that 85.7% of samples had four canals, including MB2, 11.5% had two canals, and 2.8% had one canal in our study.

This is similar to Silva et al.'s research, which found that 45.09% of cases had three distinct roots, namely mesiobuccal, palatal, and distobuccal, each with one canal. However, they had a lower incidence of MB2, with just 34.32% of the samples having MB2 [21].

In three-rooted maxillary second molars, the prevalence of MB2, the most common variant in the upper jaw, was 14.2%, which is consistent with previous studies on Korean populations and other features [3,22]. This analysis found that patients between the ages of 20 and 40 had an increased likelihood of having additional MB canals, but the variances between age sets were not significant. Both the Chinese population [8] and the Caucasian population [22] have been the subject of previous in vivo research, both of which have produced comparable results. As people age, the root canals seem to have a simpler form due to the calcification of the canal's branching structures. Therefore, practitioners should pay more attention to looking for additional canals in those aged 20-40 [22].

As with the aforementioned studies [8,22], the occurrence of additional canals was independent of tooth location. The frequency of MB2 in the maxillary second molars did not differ significantly between the sexes. Consistent with prior research, MB2 was more often found in males' maxillary (upper jaw) first molars [23,24].

Seventy pairs of maxillary second molars were used to evaluate the pattern of MB2 concurrence in contralateral molars (from 35 patients). Table 4 and Table 5 indicate the prevalence of MB2 bilateral molars in contralateral molars occurring at the same time. In the bilateral second molar pairs, this contemporaneous appearance was significant (P=0.001). According to this research, if on a molar tooth MB2 canal is found, dentists should check for additional canals in the opposite molars.

This is a retrospective study so a longitudinal study based on a population will reduce the bias and the limitations of the study. The sample size is also not so large to state the exact prevalence of the canal morphology of the region.

## Conclusions

The variation of the particular root canal is of very significant importance to determine which type of canal system is prevalent in a particular population. Diagnosis of the root canal system relates directly to the success of the root canal therapy. Cone-beam computed tomography (CBCT) is a useful tool for detecting morphological changes in the root canal trench during the examination of root canal radiographs. The root canal shape of the maxillary second molar varies widely across Meerut's population, as shown by the data collected for this research. The maxillary molar examined in this research included three different Vertucci's canal types: types I, II, and V. When comparing the root structures of maxillary second molar teeth, more variation was identified in the root trench framework and mesiobuccally foundations than in the palatal or distobuccal roots. The mesiobuccal channel is an example of Vertucci's Type II trench configuration, whereas the palatal roots and distobuccal trench are examples of his Type I waterway design. Hence the research gives an idea of the canal system prevailing in the particular population so that the dentist will be more aware of the situation while doing the therapy.
